# Local overexpression of the myostatin propeptide increases glucose transporter expression and enhances skeletal muscle glucose disposal

**DOI:** 10.1152/ajpendo.00586.2013

**Published:** 2014-01-28

**Authors:** M. E. Cleasby, S. Jarmin, W. Eilers, M. Elashry, D. K. Andersen, G. Dickson, K. Foster

**Affiliations:** ^1^Department of Comparative Biomedical Sciences, Royal Veterinary College, University of London, London, United Kingdom;; ^2^School of Biological Sciences, Royal Holloway, University of London, Egham, Surrey, United Kingdom; and; ^3^School of Biological Sciences, University of Reading, Reading, Berkshire, United Kingdom

**Keywords:** hypertrophy, insulin sensitivity, glucose transporters, insulin-like growth factor-I, AMP-activated protein kinase

## Abstract

Insulin resistance (IR) in skeletal muscle is a prerequisite for type 2 diabetes and is often associated with obesity. IR also develops alongside muscle atrophy in older individuals in sarcopenic obesity. The molecular defects that underpin this syndrome are not well characterized, and there is no licensed treatment. Deletion of the transforming growth factor-β family member myostatin, or sequestration of the active peptide by overexpression of the myostatin propeptide/latency-associated peptide (ProMyo) results in both muscle hypertrophy and reduced obesity and IR. We aimed to establish whether local myostatin inhibition would have a paracrine/autocrine effect to enhance glucose disposal beyond that simply generated by increased muscle mass, and the mechanisms involved. We directly injected adeno-associated virus expressing ProMyo in right tibialis cranialis/extensor digitorum longus muscles of rats and saline in left muscles and compared the effects after 17 days. Both test muscles were increased in size (by 7 and 11%) and showed increased radiolabeled 2-deoxyglucose uptake (26 and 47%) and glycogen storage (28 and 41%) per unit mass during an intraperitoneal glucose tolerance test. This was likely mediated through increased membrane protein levels of GLUT1 (19% higher) and GLUT4 (63% higher). Interestingly, phosphorylation of phosphoinositol 3-kinase signaling intermediates and AMP-activated kinase was slightly decreased, possibly because of reduced expression of insulin-like growth factor-I in these muscles. Thus, myostatin inhibition has direct effects to enhance glucose disposal in muscle beyond that expected of hypertrophy alone, and this approach may offer potential for the therapy of IR syndromes.

insulin resistance (IR) in skeletal muscle is an essential precursor for the development of type 2 diabetes (T2D) in humans, since this tissue is responsible for a large proportion of postprandial glucose disposal. IR is frequently associated with both increasing adiposity and age, but also commonly coexists with age-associated loss of muscle mass (sarcopenia) in a syndrome referred to as “sarcopenic obesity.” This syndrome is a substantial cause of morbidity and mortality in the elderly (affecting 4–12% of the elderly population) (37, 47), both as a consequence of metabolic derangement but also because of frailty-related accidents and immobility. However, no licensed medications exist that are effective for sarcopenia or sarcopenic obesity; thus, it is imperative that a greater understanding is gained of the mechanisms involved in its development, such that potential therapeutic targets can be identified.

Myostatin is a member of the transforming growth factor-β (TGF-β) family of secreted proteins that is almost exclusively expressed by skeletal muscle, and this tissue is also its principal target, where it inhibits myocyte hypertrophy (24, 27). Naturally occurring mutations of myostatin have been identified in a number of species, resulting in a “double muscling” phenotype (24). Similarly, myostatin null mice and mice administered with short-hairpin RNAs targeting myostatin show markedly increased muscle mass, although postnatal myostatin inhibition results in hypertrophy only, whereas null mice demonstrate both hypertrophy and hyperplasia (30, 33). Myostatin achieves its effects through activation of the Mothers against decapentaplegic homologs (25) and phosphoinositol 3-kinase (PI3K)/protein kinase B (Akt) pathways (3, 56), the latter being the site for cross talk with prohypertrophic insulin-like growth factor-I (IGF-I) signaling in muscle (56). However, activation of the PI3K/Akt pathway also plays an important role in insulin-stimulated glucose disposal in skeletal muscle (11, 53), and this is defective in muscle IR (9). In addition, myostatin modulates activation of AMP-activated protein kinase (AMPK) in muscle (59), the key enzyme in a second pathway that plays a role in both muscle metabolism and growth (26, 29).

As might be predicted, therefore, myostatin null and mutant mice also resist diet-induced obesity and IR (17, 34, 54, 59), while conversely administration of myostatin to mice causes IR (19). In addition, myostatin mRNA is increased in muscle from *ob/ob* and high-fat diet-fed mice (2) and obese (35, 40) and diabetic (39) humans, but, significantly, this change is not reflected in altered plasma levels in the latter group (6). Thus, it is as yet unclear whether myostatin's beneficial effects on metabolism are due purely to an increase in muscle mass that increases the capacity for insulin-stimulated glucose disposal, through an effect on muscle metabolism that is additional to this, or indeed whether the main beneficial effects are exerted primarily in other tissues. However, it is clear that myostatin represents a key potential molecular mediator of the pathogenesis of IR and sarcopenic syndromes, including sarcopenic obesity.

Active myostatin is generated by cleavage of a precursor protein and subsequent dimerization. The myostatin propeptide, containing the NH_2_-terminal latency-associated peptide (ProMyo) only, sequesters the myostatin dimer and prevents its binding to the activin IIB receptor (ActRIIB) (28). Mice overexpressing the propeptide show both increased muscle fiber size and number (28) and resistance to diet-induced obesity (60), whereas we have previously shown that intravenous administration of adeno-associated virus (AAV)-8-myostatin propeptide (ProMyoAAV) leads to a generalized dose-dependent increase in muscle mass (12, 32). However, studies using whole body genetic manipulations are unable to delineate the importance of the autocrine/paracrine effects of myostatin on insulin sensitivity in vivo. In this study we overexpressed ProMyo in a single muscle group to establish whether this would be sufficient to enhance insulin-stimulated glucose disposal in a tissue-autonomous fashion and whether any increase would be proportional to the enhanced muscle mass, or be the result of an additional effect of myostatin inhibition on muscle signaling or metabolism.

## MATERIALS AND METHODS

### 

#### Materials.

General reagents were supplied by Sigma-Aldrich (Gillingham, Dorset, UK). pY608-insulin receptor substrate (IRS) 1 antibody was from Biosource International (Camarillo, CA), total IRS1, total glycogen synthase kinase (GSK) 3α/β, and GLUT1 antibodies from Millipore (Billerica, MA), caveolin-1 antibody from Santa Cruz Biotechnology (Santa Cruz, CA), actin and desmin antibodies from Sigma, and all others from Cell Signaling Technology (Beverly, MA).

#### Preparation of viral vector.

The ProMyoAAV used was as previously described (12). The murine propeptide sequence used shows 99% homology with that of rat, whereas the mature peptide shares 100% homology in its amino acid sequence. HEK293T cells were cultured in roller bottles in Dulbecco's modified Eagle's medium, supplemented with 10% (vol/vol) fetal bovine serum and incubated at 37°C, 5% CO_2_. Recombinant pseudotyped AAV2/8 vector stocks were generated by using polyethylenimine and transfection with the pDD345-ProMyoFc (12) and pDP8 (46) plasmids, at a molar ratio of 1:1 in HEK293T cells. After 72 h incubation, cells were lysed, and particles were purified by using iodixanol (Sigma-Aldrich) step-gradient ultracentrifugation. The number of vector genomes was determined relative to a plasmid DNA standard using dot-blot hybridization.

#### Animals.

Male Wistar rats were obtained from Charles River (Margate, UK). Animals were maintained at 22 ± 0.5°C under a 12:12-h day-night cycle, were fed a standard maintenance chow diet (Special Diet Services, LBS Biotechnology, Horley, Surrey, UK) ad libitum, and acclimatized to their new surroundings for 1 wk. Subsequently, under brief isofluorane anesthesia, ∼7 × 10^11^ vector genomes of ProMyoAAV were injected percutaneously in 2 × 125 μl sterile saline in the right tibialis cranialis (TC) and extensor digitorum longus (EDL) muscles of ∼150-g rats, with equivalent volumes of saline being injected in the contralateral muscle group as control.

Seventeen days later, rats were starved overnight, and insulin-stimulated glucose clearance in paired muscles was measured using an intraperitoneal glucose tolerance test (IPGTT), combined with administration of 2-[1,2-^3^H(*N*)]-deoxy-d-glucose (Perkin-Elmer; Seer Green, Bucks, UK) tracer (11). Approximately 5 MBq were administered per rat in 50% glucose/0.9% saline at 2 g/kg body wt. Blood was collected by tail nick at 0, 15, 30, 60, and 90 min postinjection, and blood glucose was measured immediately using an Accu-Check Advantage meter (Roche Diagnostics, Burgess Hill, West Sussex, UK). At the end of the IPGTT, rats were killed by injection of pentobarbitone and their muscles rapidly dissected and weighed. Portions of each TC muscle were mounted in OCT compound (Sakura Finetech, Alphen aan den Rijn, Netherlands) and frozen in liquid nitrogen-cooled isopentane or freeze-clamped and stored at −80°C.

All experimental procedures were approved by the Royal Veterinary College's Ethics committee and were carried out under United Kingdom Home Office license to comply with the Animals (Scientific Procedures) Act 1986.

#### Glucose clearance into muscle.

Fresh plasma was deproteinized, its radioactivity was determined for each time point by liquid scintillation counting in Ultima Gold XR (Perkin-Elmer) on an LS6500 Multipurpose scintillation counter (Beckman Coulter, High Wycombe, UK), and the area under the curve (AUC) was calculated (Sigma Plot version 11; Systat Software, Chicago, IL). Powdered muscle was homogenized in water (Ultra-Turrax; IKA, Staufen, Germany), and the phosphorylated deoxyglucose was separated in each homogenate by passing through an anion exchange resin (Bio-Rad Laboratories, Hemel Hempstead, UK) before β-scintillation counting. The AUC and the disintegrations per minute per unit mass of muscle were used to calculate the clearance of deoxyglucose into each muscle. Plasma insulin was determined using a Rat Insulin Radioimmunoassay kit (Millipore). Triglyceride and glycogen were extracted from muscles and quantified as previously described (41).

#### Determination of muscle fiber type distribution.

Fiber type distribution was determined by simultaneous immunostaining for myosin heavy chain (MHC) isoforms type I, IIa, and IIb of 10-μm cryosections. Transverse sections of TC muscles were air-dried and blocked in 5% fetal calf serum (vol/vol) in phosphate-buffered saline containing 0.05% Triton X-100. MHC type I, IIa, and IIb fibers were identified using A4.840 IgM, A4.74 IgG, and BF-F3 IgM antibodies, respectively (Developmental Studies Hybridoma Bank, University of Iowa), in sections also immunostained with anti-laminin antibody (no. L9393, 1:100; Sigma). Type IIX fibers were identified by lack of immunostaining using these MHC antibodies. Primary antibodies were visualized using Alexa Fluor 488 (A11029, 1:200; Molecular Probes, Invitrogen, Paisley, UK) and Alexa Fluor 633 (Molecular Probes A21046, 1:200) secondary antibodies.

#### Muscle lysates, SDS-PAGE, immunoblotting.

Protein expression and phosphorylation of molecules present in muscle were assessed by SDS-PAGE and quantification of Western blots, typically in duplicate. Whole muscle lysates were prepared as previously described (41). A crude membrane preparation was used for determination of glucose transporter proteins, derived by homogenization using 20 mm HEPES, pH 7.4, 1 mm EDTA, and 250 mm sucrose, containing 25 μg/ml leupeptin, 10 μg/ml aprotinin, 2 mmol/l sodium orthovanadate, 10 mmol/l NaF, 2.5 mM sodium pyrophosphate, and 1 mmol/l polymethylsulfonyl fluoride (HES buffer) in a Dounce homogenizer, followed by 700 *g* 4°C centrifugation for 10 min, centrifugation of the supernatant at 100,000 *g* for 70 min at 4°C, and resuspension of the pellet in HES buffer plus protease/phosphatase inhibitors and 2% sodium dodecyl sulfate.

Protein content of lysate supernatants or membrane fractions was quantified using the bicinchoninic acid method (Pierce Biotechnology, Rockford, IL) using a BSA standard, normalized to the lowest concentration and denatured in Laemmli buffer for 10 min at 65°C. Aliquots containing 15–80 μg protein were resolved by SDS-PAGE, electrotransferred, and immunoblotted as previously described (10). Specific bands were detected using chemoluminescence (Western Lightning Plus; Perkin Elmer) on Fuji Super RX film (Bedford, UK), scanned and quantified using Image J software (NIH, Bethesda, MD). Data for specific protein/phosphoprotein content of whole muscle lysates were normalized to the geometric mean of the protein levels of β-actin, desmin, and glyceraldehyde 3-phosphate dehydrogenase in each lysate and then to the mean of the control muscle values.

#### Real-time PCR assay.

TC muscles were powdered under liquid nitrogen and homogenized using an Ultra-Turrax (IKA) in Trizol (Invitrogen). Extracted total RNA concentration and purity were assessed using a Nanodrop 1000 (Wilmington, DE), and integrity was confirmed by agarose gel electrophoresis. cDNA was generated using an Omniscript kit (Qiagen, Crawley, UK) after genomic DNA digestion. Real-time PCR analysis was performed using Fast Start SYBR Green Reagent (Roche Diagnostics) on a Chromo4 detector (Bio-Rad), with normalization to ROX fluorescence. Reaction mixtures contained 20 ng of cDNA and 1.5 μM each primer and were subjected to a 10-min hot start, followed by 40 cycles of 15 s at 95°C, 30 s at 55–58°C, and 30 s at 72°C, with a final 5 min extension. Primer pairs (Invitrogen) were designed using Primer 3 (http://biotools.umassmed.edu/bioapps/primer3_www.cgi) and are listed, together with the optimized PCR conditions for each, as Table 1. The relative abundance of cDNAs was quantified as previously described (43). Results are quoted after normalization to the geometric mean of mRNA levels of cyclophilin, 18S, and 36B4 expression for each muscle. Generation of appropriate products was confirmed by melting curve analysis and agarose gel electrophoresis.

**Table 1.** Primer pair sequences and annealing temperatures for real-time PCR assays of skeletal muscle mRNA expression levels

mRNA Target	Accession No.	Forward Primer	Reverse Primer	Annealing Temperature, °C
Cyclophilin	NM_017101.1	CTGGCATCTTGTCCATGG	GCTCCATGGCTTCCACAA	58
18S	NR_046237.1	GGAGAGGGAGCCTGAGAAAC	CAATTACAGGGCCTCGAAAG	55
36B4	NM_007475.5	CGACCTGGAAGTCCAACTAC	ATCTGCTGCATCTGCTTG	55
IGF-I	NM_001082477.2	CGGAGCTGTGATCTGAGGA	GTCTTGGGCATGTCAGTGTG	55
IGF-I Rec	NM_010513.2	TGACATCCGCAACGACTATC	TTGGAGATGAGCAGGATGTG	58
FOXO3	NM_019740.2	CCATGGACAACAGCAACAAG	TGGCGTTGGAATTGGTG	58
PGC1α	NM_031347.1	ACAGCTTTCTGGGTGGATTG	CGCTAGCAAGTTTGCCTCAT	55
p65	NM_199267.2	ATGCTGATGGAGTACCCTGA	GAGAAGTCCATGTCCGCAAT	55
Activin IIBR	NM_007397.2	CTACGACAGGCAGGAGTGTG	TGGCTCGTACGTGACTTCTG	55
LTBP3	NM_008520.2	AACTGCTATCCTGGCTACCG	TTTTCACATTTGCCATCAGG	55
MURF1	NM_080903.1	AGTGTGCCAACGACATCTTC	CCGGTCCATGATCACTTCAT	55
Atrogin1	NM_133521.1	GACTTCTCGACTGCCATCCT	TGAAGTTCTTTTGGGCGATG	55
Myostatin Propeptide	NM_010834.2	GGCACTGGTATTTGGCAGAG	GTCCTGGGAAGGTTACAGCA	55
Mighty	NM_001030054.1	GAAAGCCAATTCTCCCTCCTC	CCCACTTGTCGAAGGGTAAA	55

IGF-I, insulin-like growth factor-I; IGF-I Rec, IGF-I receptor; FOXO3, forkhead box O3; PGC1a, peroxisome proliferator-activated receptor coactivator 1α; p65, nuclear factor-κB p65 subunit; activin IIBR, activin IIB receptor; LTBP3, latent transforming growth factor-β-binding protein; MURF1, muscle ring finger protein 1.

#### Statistics.

Data are quoted as means ± SE. Comparisons between treated and control muscles in the same animal were made using Student's paired *t*-test. Analyses were conducted using Sigma Plot version 11, with *P* < 0.05 regarded as significant.

## RESULTS

### 

#### Local overexpression of ProMyo results in localized muscular hypertrophy but no change in muscle fiber type distribution.

**Fig. 1.**
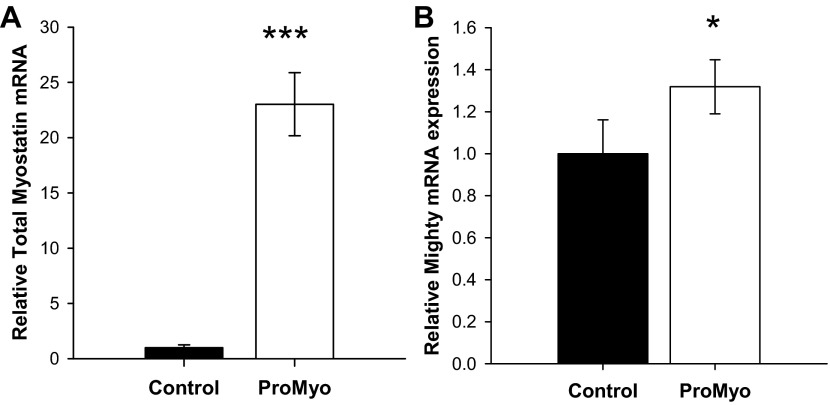
Local injection of adeno-associated virus (AAV)-8-myostatin propeptide (ProMyoAAV) increases expression of muscle myostatin propeptide (ProMyo) and akirin-1. After injection of AAV expressing ProMyo (17 days), ProMyo (*A*) and akirin-1/mighty mRNA (*B*), measured using real-time PCR, were increased in test vs. control tibialis cranialis (TC) muscles. Data are means ± SE (*n* = 5–12). Paired *t*-testing: *P < 0.01 and ***P < 0.001 vs. control.

**Fig. 2.**
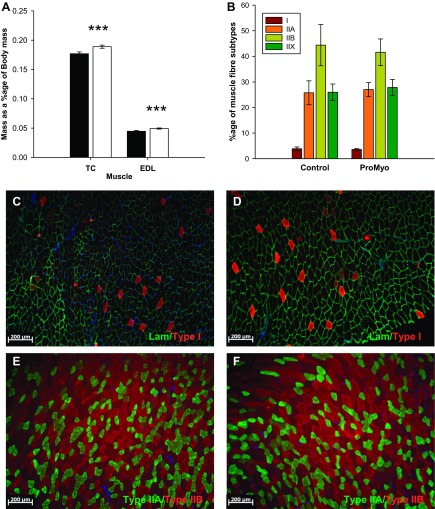
Reduced local myostatin peptide results in specific muscle hypertrophy. *A*: 17 days posttreatment there were increases in mass of both test TC and extensor digitorum longus (EDL) muscles vs. paired controls (*n* = 12). However, no differences in muscle fiber type distribution were identified in transverse TC muscle sections by immunostaining for myosin heavy chain isoforms. *B*: summary of percentage fiber type composition (*n* = 5; brown, type I; orange, type IIA; olive, type IIB; green, type IIX). Representative photomicrographs from control muscle (*C*) and ProMyoAAV-treated muscle (*D*) immunostained green for laminin and red for type I fibers and control muscle (*E*) and ProMyoAAV-treated muscle (*F*) immunostained green for type IIA and red for type IIB fibers. Filled bars, control; open bars, test muscle. Data are means ± SE. Paired *t*-testing: ***P < 0.001 vs. control.

After injection of ProMyoAAV (17 days), ProMyo mRNA was markedly increased in test vs. paired control muscles (by 23-fold, *P* < 0.001; Fig. 1*A*), consistent with a large expected reduction in the availability of the mature peptide (28). This resulted in the expected increase in expression of mighty/akirin-1, a downstream mediator of myostatin action (Fig. 1*B*) (31), and highly consistent increases in mass of both test TC and EDL muscles over control muscles (by a mean of 6.9 and 10.5%, respectively; both *P* < 0.001; Fig. 2*A*), despite the relatively short period that had elapsed since the treatment. No effect of ProMyo overexpression was observed on muscle fiber type distribution (percentage of type I, IIA, IIB, and IIX fibers per transverse section; Fig. 2, *B*–*F*) at this time point.

#### Local overexpression of ProMyo results in an elevation in glucose disposal above that expected purely as a result of the increased muscle mass.

**Fig. 3.**
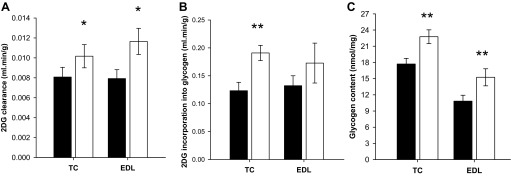
AAV-mediated local overexpression of ProMyo results in an elevation in glucose disposal above that expected by the increased muscle size. After 17 days, 2-deoxyglucose (2-DG) tracer uptake and incorporation into glycogen and lipids were measured in paired TC muscles of overnight-starved rats as part of an intraperitoneal glucose tolerance test (IPGTT). Glycogen and triglyceride content of powdered muscle from killed rats were also measured. Glucose uptake (*A*), incorporation of the label into glycogen (*B*), and glycogen content (*C*) were all increased in both TC and EDL muscles as a result of myostatin inhibition. Paired *t*-testing: *P < 0.05 and **P < 0.01 vs. the equivalent contralateral control muscle. Data are means ± SE (*n* = 6–12). Filled bars, control; open bars, test muscle. Triglyceride storage and incorporation of tracer into triglyceride during the IPGTT were unchanged by the manipulation (data not shown).

To establish whether myostatin inhibition altered acute glucose disposal, rats were subjected to an IPGTT, combined with administration of radiolabeled 2-deoxyglucose to track glucose disposal. This resulted in a typical IPGTT plasma glucose curve and an increase in plasma insulin from a basal level of 121 ± 36 to 348 ± 53 pmol/l 30 min after intraperitoneal glucose administration, consistent with the expected effect of a glucose load to cause increased insulin secretion. Clearance of deoxyglucose into test TC and EDL muscles was increased by 26 and 47%, respectively (*P* = 0.020 and 0.024; Fig. 3*A*), during the IPGTT, whereas glycogen synthesis was also increased during this period (by 55% in TCs, *P* = 0.002; not significant in EDLs; Fig. 3*B*), as demonstrated by increased incorporation of the radioactive tracer in the glycogen molecule. In addition to this acute effect on glucose disposal, ProMyo overexpression also resulted in increased glycogen content of the muscles (by 28% in TCs, *P* = 0.008 and by 41% in EDLs, *P* = 0.007; Fig. 3*C*), likely reflecting a sustained effect over the full period of the experiment.

Because these measurements are all expressed per unit mass of muscle and muscle mass was also increased, it is clear that the total glucose disposal by the treated muscles was markedly enhanced by the treatment. For example, a simple extrapolation from these findings would suggest that the mean total increase in glucose uptake (increase in muscle mass multiplied by increase in glucose uptake per unit mass) by test TCs and EDLs would be ∼35 and ∼62%, respectively.

Triglyceride content and incorporation of radiolabel into triglycerides during the IPGTT were also compared in test and control EDL muscles, but there were no effects of ProMyoAAV injection (data not shown).

#### Increased glucose disposal is associated with increases in glucose transporters.

**Fig. 4.**
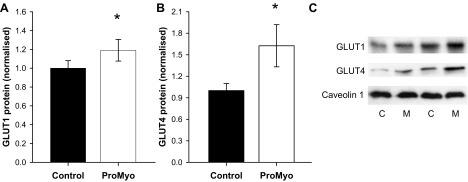
Increased glucose disposal is accompanied by increases in glucose transporter expression. Membrane-enriched TC muscle lysates were prepared and used in Western immunoblotting for glucose transporters, as described in materials and methods. Levels of both GLUT1 (*A*) and especially GLUT4 (*B*) were increased by local myostatin silencing. *C*: summary data, normalized to control levels (means ± SE; *n* = 12), are accompanied by representative blots for each transporter and for the membrane marker caveolin-1, levels of which were unchanged by the manipulation in ProMyo-treated (M) vs. control (C) muscles. *P < 0.05 vs. the equivalent contralateral control muscle.

To establish whether the increase in glucose disposal caused by ProMyoAAV administration might occur secondary to effects on glucose transporter number, we measured protein levels of GLUT1 and GLUT4 in membrane-enriched lysate fractions from paired muscles. Although levels of a membrane marker protein, caveolin-1, were unchanged by the manipulation, both GLUT1 and especially GLUT4 were increased in test muscles, by 19 and 63%, respectively (*P* = 0.031 and 0.043; Fig. 4). These data are consistent with increased capacity for basal and especially insulin-stimulated glucose uptake into propeptide-overexpressing muscles, which likely mediates the observed effect on glucose disposal.

#### Increased glucose disposal occurs despite a modest reduction in activation of the PI3K signaling pathway.

**Fig. 5.**
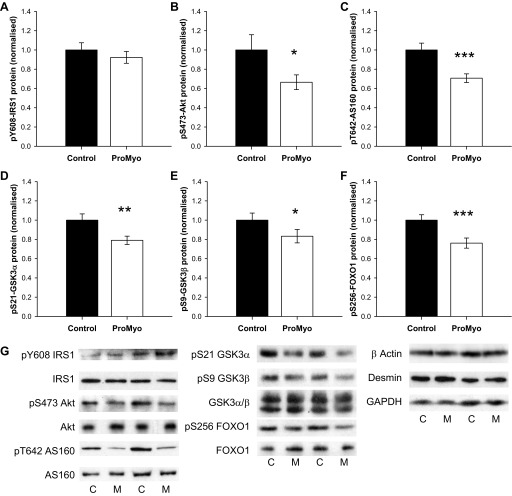
Increased glucose disposal occurs despite a modest reduction in activation of the phosphoinositol 3-kinase (PI3-kinase) signaling pathway. Paired TC muscle lysates were generated in RIPA buffer and used in Western immunoblotting for phosphorylated and total PI3-kinase signaling intermediates. Effects of myostatin silencing on phosphorylation of insulin receptor substrate 1 (IRS1, pY608) (*A*), protein kinase B (Akt, pS473) (*B*), Akt substrate of 160 kDa (AS160, pT642) (*C*), glycogen synthase kinase (GSK) 3α (pS21) (*D*), GSK3β (pS9) (*E*), and forkhead box O (FOXO) 1 (pS256) (*F*) normalized to the geometric mean of 3 housekeeping proteins [β-actin, desmin, and glyceraldehyde-3-phosphate dehydrogenase (GAPDH)] and to the contralateral control muscle. Phosphorylation of all of these proteins except IRS1 was increased by the manipulation, whereas total levels of proteins of interest and housekeeping proteins were unchanged. *G*: typical immunoblots for each protein/phosphoprotein are shown for ProMyo-treated and control muscles. *P < 0.05, **P < 0.01, and ***P < 0.001 vs. paired control. Data are means ± SE (*n* = 11–12).

Translocation of GLUT4 to the plasma membrane from its intracellular depot is required for insulin-stimulated glucose uptake by muscle cells, involving inhibition of the GTPase activity of the Rab-GTPase-activating protein Akt substrate of 160 kDa (AS160/TBC1D4) (7), whereas GSK3β-mediated disinhibition of glycogen synthase is necessary for glycogen synthesis. Both of these events rely on activation of the PI3K/Akt signaling pathway and activation of the serine/threonine kinase Akt. Thus, to establish whether activation of the PI3K/Akt pathway was also integral to the ProMyoAAV-induced increase in muscle glucose disposal, we analyzed phosphorylation and total protein levels of key signaling intermediates in muscle lysates by Western blotting. Although phosphorylation of IRS1 at Y608 was unchanged by the manipulation (Fig. 5*A*), phosphorylation of Akt at S473 was in fact decreased (by 33%, *P* = 0.022; Fig. 5*B*), indicative of reduced kinase activity. This unexpected reduction was accompanied by modest but consistent reductions in phosphorylation at target residues of all four of the Akt substrates assessed. AS160 phosphorylation was reduced by 29% at T642 (*P* < 0.001; Fig. 5*C*), which would be consistent with increased retention of GLUT4-carrying vesicles intracellularly. Similarly, phosphorylation of GSK3α and -β was also reduced (by 21 and 17%, *P* = 0.006 and 0.031; Fig. 5, *D* and *E*), which would be expected to impair glycogen synthase activation. In addition, S256 phosphorylation of forkhead box protein O1 (FoxO1) was also reduced (by 23%, *P* < 0.001; Fig. 5*F*), suggesting increased nuclear localization of this transcription factor. Total protein levels of all of the signaling intermediates were not altered by the manipulation. Thus, ProMyo overexpression results in reduced flux through the PI3K/Akt pathway distal to IRS1, which would not explain the observed effects on glucose disposal, but may instead be a secondary effect of another consequence of myostatin inhibition.

#### Local myostatin inhibition causes a reduction in activation of the AMPK signaling pathway in skeletal muscle.

**Fig. 6.**
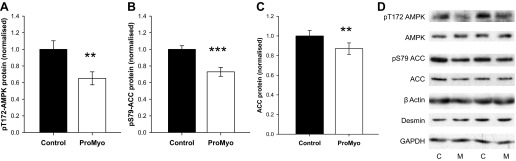
Local myostatin inhibition causes a reduction in activation of the AMP-activated protein kinase (AMPK) signaling pathway in skeletal muscle. Paired TC muscle lysates were generated in RIPA buffer and used in Western immunoblotting for phosphorylated and total AMPK and its downstream substrate acetyl-CoA carboxylase (ACC). Phosphorylation of both AMPK (pT172) (*A*) and ACC (pS79) (*B*) was reduced, as were total protein levels of ACC (*C*). Total AMPK protein levels were unchanged. Data were normalized to the geometric mean of 3 housekeeping proteins (β-actin, desmin, and GAPDH) and to the contralateral control muscle. *D*: typical immunoblots for each protein/phosphoprotein are shown for ProMyo-treated and control muscles. **P < 0.01 and ***P < 0.001 vs. paired control. Data are means ± SE (*n* = 12).

Because activation of the AMPK pathway provides an alternative stimulus for increased glucose disposal and AMPK phosphorylation and expression were reduced in myostatin null mice (58), we assessed activating phosphorylation of AMPK at T172 and phosphorylation of its substrate acetyl-CoA carboxylase (ACC) at the AMPK target residue S79. However, again, we found that phosphorylation of both enzymes was reduced by myostatin inhibition (AMPK by 35%, *P* = 0.003, Fig. 6*A*; ACC by 24%, *P* < 0.001, Fig. 6*B*). Although total AMPK protein was unaffected, these reductions in phosphorylation were also accompanied by a mean 13% reduction in ACC total protein (*P* = 0.009; Fig. 6*C*). Thus, our data rule out AMPK activation as an explanation for the ProMyo-induced increase in glucose disposal we have observed.

#### Myostatin blockade is associated with reduced expression of selected mediators of muscle size and metabolism.

**Fig. 7.**
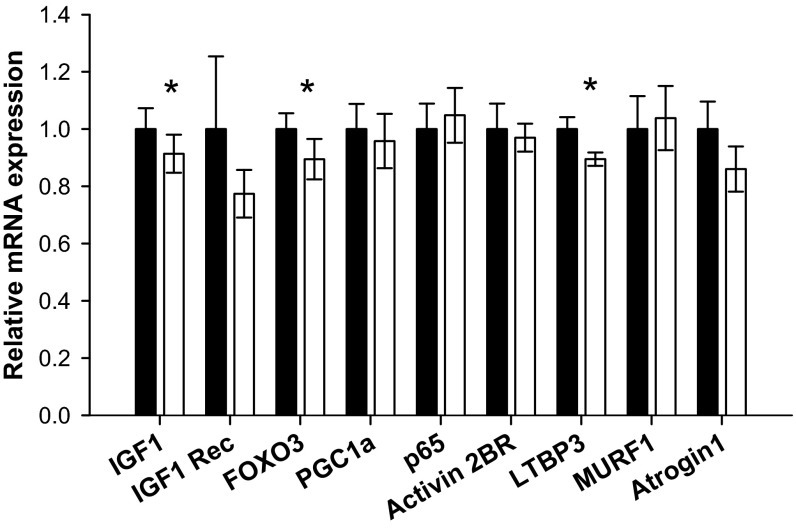
Effect of myostatin blockade on selected mediators of muscle size and metabolism. Total RNA and cDNA was prepared from paired TC muscles, and SYBR Green Real-time PCR was conducted using 20 ng of each cDNA or DNA standard in duplicate and primers as listed in materials and methods. Summary data (means ± SE; *n* = 10–12) are shown for each target mRNA of interest [insulin-like growth factor-I (IGF-I), IGF-I receptor (IGF-I Rec), FOXO3, peroxisome proliferator-activated receptor coactivator 1α (PGC1a), nuclear factor-κB p65 subunit (p65), activin IIB receptor (activin 2BR), latent transforming growth factor-β-binding protein (LTBP3), muscle ring finger protein 1 (MURF1), and atrogin 1], normalized to the geometric mean of the reference mRNAs cyclophilin, 36B4, and 18S. Filled bars, control; open bars, test muscle. *P < 0.05 vs. paired control muscles for each target.

In a further attempt to explain the ProMyo-induced phenotype of increased muscle mass and glucose disposal, we analyzed mRNA expression of a number of mediators having an impact on these parameters by real-time PCR (shown in Fig. 7). Expression of ActRIIB, the receptor for myostatin, was unchanged, but there was a 9% reduction in latent TGF-β-binding protein 3 (LTBP3) expression (*P* = 0.013), which is the most common LTBP expressed in skeletal muscle and is thought to sequester promyostatin extracellularly (4), implying that reduced transcription is likely a compensatory effect of the overexpression.

Expression of the proatrophic transcription factor nuclear factor-κB (active p65 subunit) was unchanged, but there was a small reduction in the expression of FoxO3 (11%, *P* = 0.045). Nevertheless, consistent with previous reports that myostatin does not work through promotion of protein degradative/atrophic pathways (50), expression of the E3 ubiquitin ligases muscle ring finger protein (MURF) 1 and atrogin 1 (muscle atrophy F-box protein) (5) were not altered.

With regard to prohypertrophic factors, there was no change in expression of the mitochondrial regulator peroxisome proliferator-activated receptor coactivator-1α. Although IGF-I receptor (IGF-IR) mRNA was not significantly altered, there was a small decrease in IGF-I expression (by 8%, *P* = 0.025), again presumably a compensatory change for profound myostatin inhibition. However, a local reduction in IGF-I may provide the explanation for the observed reductions in activation of the PI3K/Akt and AMPK signaling pathways in ProMyoAAV-injected muscles.

## DISCUSSION

In our study we have demonstrated that local inhibition of myostatin action in skeletal muscle for only a relatively short period is capable of generating both increased muscle mass and glucose disposal in rats. Both glucose uptake and glycogen synthesis were increased when measured per unit muscle mass. Thus, the combination of simultaneous increases in muscle mass and glucose disposal would imply that there was a marked increase in total glucose disposal in the muscle group tested. The fact that this enhancement was present just 17 days after injection of the virus suggests that, with additional time, a greater magnitude of effect could be expected because of further accretion of muscle tissue. We have also observed a greater than proportional increase in glucose disposal both as a result of acute muscle Akt-2 overexpression (11) and universal overexpression of urocortin-3 (20), implying the possibility of a more general mechanism worthy of deeper investigation.

The increased glucose disposal was associated with increased GLUT1 and GLUT4 glucose transporter content, implying an increased capacity for both basal and insulin-stimulated glucose uptake (the latter being of more relevance during the IPGTT, when total levels of plasma membrane GLUT4 would be much higher than those of GLUT1). The results of transgenic overexpression of GLUT4 in muscle (51) suggest that this change would be sufficient to explain the improved glucose disposal observed here, and indeed both basal and insulin-stimulated glucose disposal and GLUT4 protein were elevated in an analogous in vivo model of deficient myostatin action, the myostatin null mouse (58), while muscle glycogen content was also increased by treatment with an ActRIIB inhibitor (14). We can speculate that the increased glycogen synthesis is likely a result of allosteric stimulation of glycogen synthase activity by rising levels of glucose 6-phosphate, secondary to increased glucose transport, since we found GSK3β phosphorylation at Ser^9^ to be reduced. Furthermore, because no differences in triglyceride synthesis or content were noted in propeptide-overexpressing muscles, it does not appear that the effects on glucose disposal are secondary to reduced lipid accumulation, although we do not have data regarding some of the more bioactive lipid species.

At first glance, the modest downregulation of the AMPK and PI3K/Akt signaling pathways in the ProMyo-treated muscles seems to be counterintuitive, since these pathways mediate both muscle hypertrophy and glucose disposal. Akt has the potential to regulate muscle size through several mechanisms, including via phosphorylation of both GSK3 and FoxOs. GSK3 inhibits the translation initiation factor eIF2B (44), whereas FoxOs have been shown to promote muscle atrophy through activation of the E3 ubiquitin ligases MURF1 and atrogin-1 (45, 48). In a previous study, myostatin overexpression in rats did not alter levels of expression or phosphorylation of FoxO1, or indeed expression of E3 ubiquitin ligases (3). Instead, this manipulation caused inhibition of the Akt-mTOR pathway, suggesting an impact on protein synthesis rather than degradation, an effect that was borne out here by the lack of effect of ProMyoAAV injection on MURF1 and atrogin-1. However, transgenic overexpression of FoxO1 in mouse skeletal muscle caused atrophy (21); thus, the reduction in FoxO1 phosphorylation would tend to support the hypertrophy induced by myostatin blockade in our study. Both FoxO1 (8) and FoxO3 (15) and their target genes have been shown to be regulated by AMPK as well as Akt; thus, the reduced phosphorylation/expression of these transcription factors may be secondary to reduced activation of either upstream kinase.

The observed reduction in IGF-I expression in the test muscles may at least in part explain the reduced activation of the PI3K/Akt pathway, of which it is a key activator, and also the AMPK pathway (38). This change is consistent with that observed previously in myostatin knockout mice (13, 55), although in these animals the reduction in IGF-I was accompanied by increases in IGF-IR and IGF-I-binding protein expression and/or increased IGF-II expression. Muscle-derived IGF-I is known to have important paracrine/autocrine effects (20, 42); thus, a local compensatory effect of myostatin inhibition to decrease IGF-I mRNA transcription may be implicated here. Although we did not detect a change in phosphorylation of IRS1, the usual adaptor protein recruited by the IGF-IR to activate the PI3K/Akt pathway (42), it is possible that this was a sensitivity artifact of the Western blot or that an alternative adaptor protein may have been recruited, for example, IRS2 (49). Although many publications have investigated the effects of myostatin inhibition downstream at the level of Akt and below, there is little published information on the impact of this manipulation upstream of Akt.

Injection of ProMyoAAV in a specific muscle group resulted in local effects, since a number of significant differences in muscle mass, metabolism, and signaling were demonstrated in the injected muscles. In addition, our data confirm that increased muscle mass alone is not the likely explanation for the observed beneficial effects of systemic myostatin inhibition on metabolism, which include reduced fat mass, increased “browning” of adipose tissue, and improved whole body insulin sensitivity (1, 16, 17, 34, 58–60). These effects make systemic inhibition of myostatin a potentially viable approach for the therapy of T2D, the metabolic syndrome, and sarcopenic obesity. A number of approaches aimed at reducing myostatin action for the treatment of muscular dystrophies have already been considered, including gene therapy by delivery of myostatin-inhibiting genes, propeptide or small-interfering RNA using AAV or retrovirus (12, 18, 57), administration of antimyostatin blocking antibodies (52) or ActRIIB competitors (1, 14, 36, 59), and antisense oligonucleotide-mediated exon skipping (22, 23). However, it is unclear as yet whether any of these therapeutic modalities will have long-term potential for the treatment of metabolic disorders, and thus more research into mechanisms and applications is warranted.

In conclusion, our study demonstrates that myostatin inhibition has autocrine/paracrine effects to enhance glucose uptake and glycogen synthesis in skeletal muscle of rats, which are likely mediated through increased membrane levels of GLUT1 and GLUT4 glucose transporters. This effect occurs in tandem with increased muscle mass, but is additional to it, thus magnifying the overall effect. These findings support the potential utility of strategies aimed at inhibiting skeletal muscle myostatin action in the treatment of metabolic disorders, including T2D and sarcopenic obesity.

## GRANTS

This work was funded by the Wellcome Trust (a University Award to M. E. Cleasby) and Pfizer (previously Wyeth).

## DISCLOSURES

No conflicts of interest, financial or otherwise, are declared by the authors.

## AUTHOR CONTRIBUTIONS

Author contributions: M.E.C., G.D., and K.F. conception and design of research; M.E.C., S.J., W.E., M.E., and D.K.A. performed experiments; M.E.C. and K.F. analyzed data; M.E.C. interpreted results of experiments; M.E.C. prepared figures; M.E.C. drafted manuscript; M.E.C., S.J., G.D., and K.F. edited and revised manuscript; M.E.C., S.J., W.E., M.E., D.K.A., G.D., and K.F. approved final version of manuscript.
